# 

**Published:** 1892-07

**Authors:** 


### TABLE OF CONTENTS.

**ORIGINAL COMMUNICATIONS.** PAGE

Crown- and Bridge-Work. By Dr. C. M. Richmond, New York . . . . 485
The Factors concerned in Dental Deterioration. By L. C. F. Hugo, D.D.S., Washington, D.C. . . . . . . . . . . . . . . . . . . . . 489
The Teeth and the Feet. By Thomas C. Stellwagen, M.D., D.D.S. . . 502
Dental Litigation, Past and Present. By Joseph Head, M.D., D.D.S. . 510
Some Observations on the Use of Copper Amalgam. By Dr. J. Allen Osmun, Newark, N. J. . . . . . . . . . . . . . . . . . . . . 515
Copper Amalgam. By Dr. S. B. Palmer, Syracuse, N. Y. . . . . . . . 521
Amalgam as a Filling-Material. By William P. Cooke, D.M.D. . . . 522

**REPORTS OF SOCIETY MEETINGS.**

New York Odontological Society . . . . . . . . . . . . . . . . . 527
Odontological Society of Pennsylvania . . . . . . . . . . . . . . 538
American Academy of Dental Science . . . . . . . . . . . . . . . 541

**EDITORIAL.**

Honorary Degrees . . . . . . . . . . . . . . . . . . . . . . . 543
The Conventions . . . . . . . . . . . . . . . . . . . . . . . . 546
The Wilcox Bill . . . . . . . . . . . . . . . . . . . . . . . . 547
Dr. Josef Arkövy . . . . . . . . . . . . . . . . . . . . . . . 547

**BIBLIOGRAPHY** . . . . . . . . . . . . . . . . . . . . . . . . 548

**OBITUARY.**

Dr. Daniel Dwyer . . . . . . . . . . . . . . . . . . . . . . . 551
Dr. John Allen . . . . . . . . . . . . . . . . . . . . . . . . 552
Professor Carl Sauer . . . . . . . . . . . . . . . . . . . . . 552

**CURRENT NEWS.**

The Wilcox Bill . . . . . . . . . . . . . . . . . . . . . . . . 553
Meeting of the Pennsylvania State Dental Society and the Joint Meeting of the Pennsylvania and the New Jersey State Dental Societies . . . . . . 558
American Dental Association . . . . . . . . . . . . . . . . . . 558
New Jersey Examinations . . . . . . . . . . . . . . . . . . . . 558
National Association of Dental Faculties . . . . . . . . . . . . 559
First District Dental Society, State of New York . . . . . . . . 559
Minnesota State Dental Association . . . . . . . . . . . . . . . 559
South Carolina State Dental Association . . . . . . . . . . . . 560
Patents . . . . . . . . . . . . . . . . . . . . . . . . . . . . 560
Connecticut State Dental Association . . . . . . . . . . . . . . 561
Illinois State Dental Society . . . . . . . . . . . . . . . . . 561
Buffalo Dental Association . . . . . . . . . . . . . . . . . . 561
Commencement . . . . . . . . . . . . . . . . . . . . . . . . . 562

**SELECTIONS.**

Hand Disinfection . . . . . . . . . . . . . . . . . . . . . . . 562
Europhen . . . . . . . . . . . . . . . . . . . . . . . . . . . 563
To deodorize Iodoform . . . . . . . . . . . . . . . . . . . . . 564
Peroxide of Hydrogen as a Disinfectant of Water . . . . . . . . . 564

